# The Importance of Neuroimaging Follow-Up in Bilirubin-Induced Encephalopathy: A Clinical Case Review

**DOI:** 10.3390/brainsci15060539

**Published:** 2025-05-22

**Authors:** Martina Resaz, Alessia Pepe, Domenico Tortora, Andrea Rossi, Luca Antonio Ramenghi, Andrea Calandrino

**Affiliations:** 1 Neuroradiology Unit, Department of Services, IRCCS Istituto Giannina Gaslini, 16147 Genoa, Italy; martinaresaz@gaslini.org (M.R.); domenicotortora@gaslini.org (D.T.); andrearossi@gaslini.org (A.R.); 2 Department of Neuroscience, Rehabilitation, Ophthalmology, Genetics, Mother and Child Health, School of Medical and Pharmaceuticals, University of Genoa, 16132 Genoa, Italy; lucaramenghi@gaslini.org; 3 Department of Health Science, University of Genoa, 16132 Genoa, Italy; 4 Neonatal Intensive Care Unit, Department of Maternal and Neonatal Health, IRCCS Istituto Giannina Gaslini, 16147 Genoa, Italy; andrea.calandrino@edu.unige.it

**Keywords:** hyperbilirubinemia, kernicterus, MRI, neuroimaging, newborn

## Abstract

**Introduction:** Hyperbilirubinemia in newborns can lead to kernicterus, a severe form of neonatal encephalopathy caused by bilirubin toxicity. Despite timely interventions such as exchange transfusion, kernicterus can still develop, especially in high-risk infants. MRI is crucial for detecting early and evolving signs of bilirubin-induced brain damage. **Case Report:** We report a term newborn who developed severe hyperbilirubinemia and kernicterus despite receiving exchange transfusion. The infant presented on day 3 of life with jaundice, hypotonia, and feeding difficulties and had a bilirubin level of 51 mg/dL. After exchange transfusion, bilirubin levels normalized, but neurotoxicity persisted. Initial MRI at one month showed mild T1 hyperintensity in the hippocampi with no changes in the basal ganglia. At two months, T1 hyperintensities in the hippocampi partially resolved. By six months, MRI revealed T2 hyperintensities in the globus pallidus and hippocampal atrophy, consistent with kernicterus. Magnetic resonance spectroscopy (MRS) showed reduced N-acetylaspartate (NAA) levels, indicating neuronal loss. **Discussion:** MRI is essential in monitoring bilirubin-induced brain injury. In this case, early MRI findings showed mild hippocampal T1 hyperintensity, which resolved partially. At six months, T2 hyperintensities in the globus pallidus confirmed chronic bilirubin encephalopathy. MRS demonstrated a reduction in N-acetylaspartate, indicative of neuronal loss. Susceptibility-Weighted Imaging (SWI) showed no abnormalities, likely due to the myelination process in neonates. **Conclusions:** This case highlights the importance of repeated MRI in detecting bilirubin-induced brain damage. Early neuroimaging enables timely interventions and improves long-term neurodevelopmental outcomes in infants with severe hyperbilirubinemia.

## 1. Introduction

Hyperbilirubinemia is a common condition in newborns, characterized by elevated levels of bilirubin in blood leading to jaundice [1]. The incidence of hyperbilirubinemia varies significantly between term and preterm infants. In term newborns, approximately 60% develop some degree of jaundice, though only about 5–10% reach bilirubin levels that require phototherapy, and less than 1% escalate to levels necessitating exchange transfusion [2,3]. In contrast, preterm infants are at higher risk due to immature liver function and decreased bilirubin clearance. Up to 80% of preterm neonates may develop jaundice, with about 10–15% requiring phototherapy. The proportion of preterm infants needing exchange transfusion, although higher than that for term infants, remains relatively low, typically ranging from 1 to 5% depending on gestational age and comorbidities [4]. Early detection and management are crucial to prevent complications such as kernicterus [5,6].

Kernicterus is a term that describes acute neonatal encephalopathy associated with brain toxicity due to unbound unconjugated bilirubin that crosses the blood–brain barrier. Serum bilirubin levels exceeding 25–30 mg/dL are typically associated with this condition [7].

The epidemiology of kernicterus has evolved with advancements in neonatal care. In developed countries, where routine bilirubin screening and early interventions such as phototherapy are standard practice, the incidence of kernicterus has significantly declined. It is estimated to occur in 0.5 to 1 per 100,000 live births among term infants in high-resource settings [8]. Conversely, in low- and middle-income countries, where access to timely medical care and phototherapy may be limited, the incidence is considerably higher, ranging from 1 to 4 per 1000 live births [9]. Preterm infants are particularly vulnerable due to lower thresholds for bilirubin neurotoxicity, increasing their risk of developing kernicterus even at bilirubin levels considered safe for term neonates. Early detection, vigilant monitoring, and prompt management of hyperbilirubinemia remain critical to prevent this severe, lifelong neurological condition [10].

In this paper, we describe a case of severe hyperbilirubinemia in a term newborn who, despite receiving proper exchange transfusion therapy, developed kernicterus. We provide a detailed neuroimaging analysis, comparing magnetic resonance imaging findings at different time points to illustrate the progression of brain injury and possible early detection neuroimaging markers.

## 2. Case Report

Our patient, a Caucasian male infant, was born at home by eutocic delivery at 40 weeks of gestation. Antenatal imaging and serology were unremarkable, and there was no family history of metabolic diseases. At birth, positive pressure ventilation was required within the fifth minute of life; the APGAR score was 7 at 1 min and 10 at 5 min. The birth weight was 3260 g (Average for Gestational Age (AGA), according to INeS charts). The patient was admitted to the NICU on day 3 of life for jaundice, hypotonia, hyporeactivity, and feeding difficulties. Blood exams on admission revealed significant hyperbilirubinemia (total bilirubin level: 51 mg/dL, predominantly unconjugated), blood type 0, Rhesus-positive, positive direct Coombs test (maternal blood type was A, Rhesus-positive). In addition, blood cultures were positive for *E. faecalis*, *S. aureus*, and *E. coli*, leading to a concomitant diagnosis of early-onset neonatal sepsis. Neurological examination revealed signs consistent with bilirubin-induced neurotoxicity. The infant exhibited hypotonia with diminished spontaneous movements and a weak Moro reflex. Suck and rooting reflexes were markedly impaired. There was poor feeding behavior accompanied by episodes of high-pitched crying, suggestive of irritability and potential central nervous system involvement. Cranial nerve examination was conducted through structured observation during periods of spontaneous alertness. Limited upward gaze and poor tracking were identified based on the infant’s inability to visually follow stimuli above midline and laterally, respectively, despite normal pupillary responses.

The patient underwent a blood exchange transfusion immediately after admission, reaching safe serum bilirubin levels after just one cycle. Concomitantly, broad-spectrum antibiotic therapy was initiated with complete resolution of the clinical sepsis after one week of treatment. Neurological objectivity showed improvement in the following days, with persistent difficulties in gaze control and heightened reactivity indicating partial recovery with ongoing neurological impairment. Further examinations were performed: Auditory Brainstem Response (ABR) showed no responses, indicating possible auditory pathway involvement. However, both electroencephalogram (EEG) and fundoscopy were normal, suggesting no widespread cortical or retinal abnormalities.

**Figure 1:**
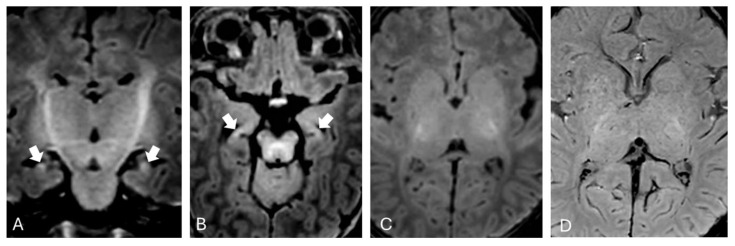
Brain MRI performed during the subacute phase. Hyperintensity in the hippocampi on coronal (**A**) and axial (**B**) T1-weighted images (arrows, **A**,**B**). No evident changes were observed in the nucleo-capsular region, either in axial T1-weighted images (**C**) or in susceptibility-weighted images (**D**).

Brain MRI was performed at one month of extrauterine life as expected from existing literature [11] and showed mild T1 hyperintensity in the hippocampi bilaterally (Figure 1).

**Figure 2:**
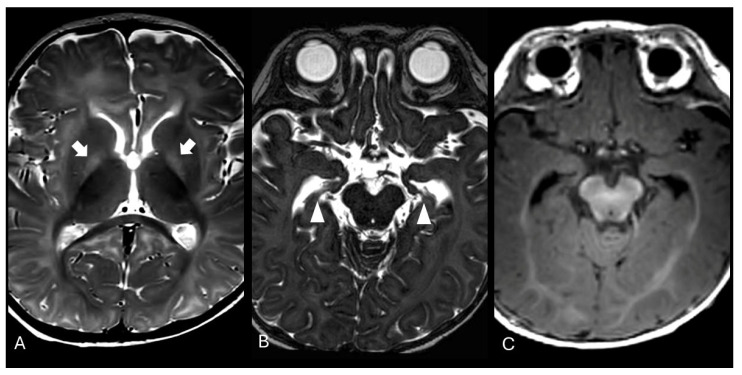
Brain MRI obtained at 6 months. (**A**) An axial T2-weighted image showing hyperintensity in globus pallidus bilaterally (arrows), a typical finding in chronic bilirubin encephalopathy; (**B**) an axial T2 DRIVE image showing tissue loss in both hippocampi (arrowheads), corresponding to the areas that previously showed T1 hyperintensity (see Figure 1) which are no longer detectable on a T1-weighted image (**C**).

The exam was repeated at two months of life, showing partial resolution of the previously described alterations, and again at six months of age, revealing T2-weighted hyperintensity in both globi pallidi and hippocampal atrophy (Figure 2). Proton magnetic resonance spectroscopy (MRS) of the basal nuclei showed a minimal reduction in N-acetylaspartate (NAA) levels. These neuroradiological findings were compatible with sequelae of kernicterus.

Clinically, the described lesions resulted in a neuropsychomotor profile characterized by strabismus, intermittent gaze, and poorly fluid and organized motor activity with dyskinetic movements. There was a slight presence of antigravity movements of the lower limbs, which were quickly exhausted. Muscle tone was within normal limits, although there was an absence of complete head control, with the ability to lift the head only briefly when placed in a prone position. The hands were clenched, with occasional spontaneous opening and flexion of the distal interphalangeal joints along with a transient grasping ability. Further investigations, including visual evoked potentials (VEPs) and fundoscopy, were within normal limits. However, ABR threshold testing revealed retrocochlear hearing loss with a conductive component, requiring the application of a cochlear implant.

Over time, the infant developed hypertonia with opisthotonus posturing, and deep tendon reflexes were exaggerated, with the presence of sustained clonus.

## 3. Discussion

Kernicterus is an uncommon but severe condition that leads to acute neonatal encephalopathy and may result in long-term neurocognitive impairments, such as sensorineural hearing loss and dyskinetic cerebral palsy [12].

Kernicterus follows a specific multiregional damage pattern, primarily affecting the globus pallidus, subthalamic nucleus, geniculate nucleus, dentate nucleus, inferior olives, nucleus gracilis and cuneatus, hippocampus, mammillary bodies, red nucleus, substantia nigra, cranial nerve nuclei, colliculi, and the cerebellar vermis [13]. The main effects of bilirubin on neurons and oligodendrocytes include apoptosis, oxidative stress, and reduced myelin synthesis, accompanied by a proinflammatory microglial reaction that initiates a metabolic cascade leading to neurotoxicity [14,15].

This pathological process explains the typical imaging signature observed in the acute phase of kernicterus. Magnetic resonance imaging (MRI) sequences, particularly T1-weighted images, show phase signal hyperintensity in the globus pallidus and subthalamic nucleus [16]. However, this hyperintensity represents only a gradient of increased signal compared to normal tissue and is not sufficiently distinctive to confirm the extent of neuronal death [17]. Over time, usually after several weeks, a fine fibrillary astrogliotic scar becomes evident, permanently marking the globus pallidus on T2-weighted MRI scans [18].

In our patient, brain MRI performed during the subacute phase revealed hyperintense signal on T1-weighted images within the hippocampi, while T2-weighted images in the same regions appeared normal. No significant alterations were observed in the basal ganglia and subthalamic nuclei on T1-weighted sequences. The absence of obvious alterations in T1-weighted sequences may be attributed to the myelination process, which makes it more difficult to distinguish pathological changes from the expected T1 hyperintensity in these regions during normal developmental processes at this age [19]. This is particularly true when using high-field imaging, which provides a better signal-to-noise ratio. Alternatively, this could be related to the onset of the “blind window” described by Gburek-Augustat et al. [18], which is typically observed around two months of life, during which no alterations are detectable on MRI.

During the follow-up at 2 months, a progressive normalization of the signal alterations in T1-weighted sequences in the hippocampal region was observed. At the 6-month follow-up, there was focal tissue loss, with no signal alterations in the T1-weighted sequences. In this last examination, the appearance of T2 hyperintensities in both globi pallidi was also noted, as classically described in the literature in the sequel of kernicterus, consistent with gliosis [20,21]. Additionally, magnetic resonance spectroscopy (MRS) demonstrated a reduction in the N-acetylaspartate peak compared to age-matched controls, suggesting neuronal and axonal loss.

The role of Susceptibility-Weighted Imaging (SWI) remains unclear. Lequin et al. [22] recently suggested it as a more specific and sensitive tool for detecting pathological alterations in kernicterus. However, in our patient, SWI remained unremarkable. In our opinion, the use of SWI to study the nucleo-capsular region in newborns and infants remains challenging due to the myelination process and the diamagnetic properties of myelin itself [23], which appears slightly hyperintense. Additionally, the presence of T1 hyperintense lesions can lead to a hyperintense signal in SWI images, due to a “T1 shine-through” effect [24,25]. Potential additional information can be obtained using Quantitative Susceptibility Mapping (QSM) due to its higher anatomical resolution compared to SWI and the possibility of quantifying the variation in magnetic susceptibility in brain tissue [26]. In this context, QSM could represent a potential auxiliary tool in early diagnosis and longitudinal monitoring of damage evolution, for instance by correlating the obtained data with blood bilirubin levels, but further studies are needed.

From a clinical point of view, our patient exhibited long-term sequelae, including hearing loss and motor impairment. This underscores the critical importance of not only monitoring bilirubin levels in neonates during hospitalization and after discharge—particularly in the presence of risk factors such as maternal–neonatal ABO incompatibility—but also ensuring close neuroimaging follow-up, even after the resolution of clinical and laboratory abnormalities. In our case, MRI performed at one and two months of age detected only slight T1 hyperintensity within the hippocampi bilaterally, while the last follow-up neuroimaging at six months of age revealed a T2 hypersignal within both globi pallidi and tissue loss in the hippocampi, a neuroimaging hallmark of chronic bilirubin encephalopathy.

## 4. Conclusions

In conclusion, it is important to emphasize that even in the absence of detectable injury on the first MRI study, close clinical and radiological follow-up is essential to identify neurological damage in the brain regions typically affected by kernicterus. In fact, even in the absence of significant clinical symptoms or in the presence of non-specific findings, as often occurs, the detection of these alterations allows for the implementation of early neuro-rehabilitative strategies aimed at reducing morbidity and long-term complications related to neurotoxicity, thereby promoting optimal neurodevelopmental outcomes.

This case also underscores the complexity of bilirubin-induced neurotoxicity and the limitations of current therapeutic approaches in fully preventing kernicterus, even when prompt and appropriate interventions such as exchange transfusion are administered.

Furthermore, we respectfully challenge the notion that Susceptibility-Weighted Imaging (SWI) is consistently a sensitive and reliable modality for detecting kernicterus-related brain injury. Our case, in agreement with contrasting reports in the literature, demonstrates that SWI may remain unremarkable in early or subtle stages of bilirubin-induced damage—particularly in neonates, where the physiological process of myelination and the diamagnetic properties of myelin can mask lesions, and where T1 shine-through artifacts may obscure interpretation.

In this context, we propose Quantitative Susceptibility Mapping (QSM) as a promising alternative. QSM provides superior anatomical specificity and enables the quantitative assessment of magnetic susceptibility, potentially allowing for early abnormality detection. While its use in clinical settings remains limited, we believe that QSM merits further investigation as a valuable tool for both early diagnosis and longitudinal monitoring in neonatal bilirubin encephalopathy, particularly in cases where SWI findings are inconclusive.

## Data Availability

Data are available upon reasonable request. The data sets generated during and/or analyzed during the current study are not publicly available but are available from the corresponding author on reasonable request.

## Abbreviations

The following abbreviations are used in this manuscript:

MRI	Magnetic Resonance Imaging
MRS	Magnetic Resonance Spectroscopy
NICU	Neonatal Intensive Care Unit
NAA	N-Acetylaspartate
SWI	Susceptibility-Weighted Imaging
ABR	Auditory Brainstem Response
EEG	Electroencephalogram
VEPs	Visual Evoked Potentials
AGA	Average for Gestational Age
QSM	Quantitative Susceptibility Mapping
